# Uncommon Causes of Chest Pain in Children: An Experience From a Tertiary Care Hospital

**DOI:** 10.7759/cureus.37203

**Published:** 2023-04-06

**Authors:** Nurul Islam, Saroj K Tripathy, Jitendriya Biswal

**Affiliations:** 1 Pediatrics, Healthworld Hospitals, Durgapur, IND; 2 Pediatrics, All India Institute of Medical Sciences, Deoghar, Deoghar, IND; 3 Psychiatry, Institute of Medical Sciences & SUM Hospital, Bhubaneswar, IND

**Keywords:** screen time, cardiac, psychogenic, children, chest pain

## Abstract

Background

Chest pain is one of the common complaints for emergency and outpatient department (OPD) visits in children and adolescents. Chest pain accounts for 0.6% of pediatric emergency visits and 2.5% of pediatric outpatient consultations. The prevalence of chest pain and the etiological factors associated with children in India is unclear. The primary objective of this study was to evaluate the etiology of chest pain in children and adolescents. The secondary objective was to describe the demographic characteristics and associated symptoms with chest pain and the outcomes of children after the intervention.

Methodology

A retrospective analysis of case records of 55 children aged between 5 and 15 years who attended the emergency or OPD of the hospital with the primary complaint of chest pain from July 1, 2019, to June 30, 2021, was done.

Results

The mean age of patients in our study was 10.75 ± 2.47 years. Of 55 children, 26 were males, and 26 were females (male-to-female ratio = 0.9). In total, 43 (78.2%) patients had screen time of more than two hours. Palpitation was recorded in 11 (20.4%) patients, whereas only four (7.3%) children had breathing difficulties. Of 55 children, 46 (83.6%) had psychogenic causes, six (10.9%) had organic reasons, and three had no identifiable cause of chest pain. Anxiety disorder (40%) and depression (21.8%) were the leading psychogenic causes of chest pain. Overall, 13 (23.6%) children had associated smartphone and internet addiction disorder. Of 55 children, 36 (63.6%) improved following an appropriate intervention. Five children had some or no improvement in chest symptoms. Finally, 15 (27.3%) children were lost to follow-up.

Conclusions

Chest pain is one of the common complaints in the pediatric age group needing referral to a pediatric cardiologist. The most common etiology associated with chest pain is often non-cardiac and psychogenic. Good patient history taking, clinical examination, and fundamental investigations are sufficient to unravel the etiology in most cases.

## Introduction

Chest pain is one of the common complaints for emergency and outpatient department (OPD) visits in children and adolescents. Although the exact prevalence of chest pain in children is unknown, it accounts for 0.6% of pediatric emergency visits and 2.5% of pediatric outpatient consultations [1]. In adults, chest pain is thought to be an important symptom of an underlying cardiac disorder. Hence, it is common for parents to feel excessively worried and uneasy when their child experiences chest pain. The etiology of chest pain in children includes both cardiac and non-cardiac causes. Non-cardiac causes are musculoskeletal, gastrointestinal, respiratory, psychogenic, and idiopathic in most patients [2-4]. Less commonly, cardiac causes such as congenital and acquired heart diseases (arrhythmia, rheumatic heart diseases, cardiomyopathies, and coronary artery abnormalities) account for 0-15% of all causes of chest pain [5].

The prevalence of chest pain and the etiological factors associated with children in India is unclear. From a recent study involving the Western population, the most common causes of chest pain were musculoskeletal (33%) and psychogenic (28.4%) [6]. Because of ethnic, cultural, social, and geographical disparity, non-cardiac causes of chest pain may have regional variations. In the absence of data from India, it is imperative on our part to study the causes of chest pain in children, which may serve as a template for better and more focused clinical management. Our study aimed to evaluate the etiology of chest pain in children and adolescents.

## Materials and methods

A retrospective analysis was conducted by reviewing patients’ past medical records from the HealthWorld Hospital, Durgapur, and West Bengal, India hospital database. Children aged 5-15 years with a primary complaint of chest pain who attended the OPD or emergency department of the hospital from July 1, 2019, to June 30, 2021, were considered for inclusion in this study. Ethical clearance was obtained from the institutional ethical committee before the study. All children underwent thorough history taking, physical examination, and electrocardiography (ECG). Echocardiography was done in high-risk cases by a trained pediatric cardiologist. Any obvious abnormality in physical examination such as joint laxity, skeletal deformity (kyphoscoliosis), and chest asymmetry was noted. Any additional investigations such as complete hemogram, lipid profile, chest X-ray, exercise stress test, upper gastrointestinal endoscopy, pulmonary function test, and angiography were done as required based on the clinical scenario and examination findings. The family’s socioeconomic status was categorized into upper, middle, and lower based on the modified Kuppuswamy scale [7]. Broadly, chest pain was categorized into organic, psychogenic, and idiopathic. Organic causes of chest pain include cardiac, musculoskeletal, respiratory, and gastrointestinal causes. A psychiatrist evaluated all children with non-organic causes of chest pain, and the diagnosis was made according to the Diagnostic Statistical Manual of Mental Disorders Fifth Edition (DSM-5) criteria. Chest pain in which neither organic nor psychogenic cause could be identified was categorized as idiopathic [8]. A therapeutic counseling process was conducted as part of routine treatment. A brief cognitive behavioral therapy model was adopted, focussing on stress management and coping skills in patients with psychogenic conditions. Treatment of organic cause was directed against the underlying cause, and symptomatic treatment was provided for idiopathic chest pain.

Data were collected from the hospital records of individual patients. Baseline data such as age, sex, religion, education, anthropometry, and residence were recorded. The number of siblings, parental education, employment, and family income were noted. The child’s habits such as food habits, screen time, sleep habits, outdoor playtime, and associated symptoms such as palpitation, breathing difficulty, and abdominal pain were noted. The etiology of chest pain was recorded after a thorough evaluation. Follow-up records of patients for at least six months were reviewed when available, and details of outcomes were observed. Data collected were entered in MS Excel. Baseline variables were expressed in percentages and mean ± SD. Different etiology and factors associated with the chest were expressed in percentages. The outcome of chest pain was expressed in percentages. Fisher’s exact test compared the etiology of chest pain in males and females. P-value ≤0.05 was considered significant. Data collected were analyzed by MS Excel and GraphPad Prism 9 software.

## Results

A total of 55 children and adolescents between 5 and 15 years of age met the inclusion criteria and were included in this study. The mean age of patients in our study was 10.75 ± 2.47 years. Of 55 children, 26 were males, and 26 were females (male-to-female ratio = 0.9). Around 73% of children belonged to Hindu families, and 28% belonged to Muslim families. Most patients (65.5%) with chest pain were from highly educated families. Both parents of these children had graduation or higher qualification. Per the modified Kuppuswamy scale, 45 (81.8%) chest pain patients belonged to upper and middle socioeconomic families. Only 18.2% of children complaining of chest pain were from families with lower socioeconomic status. The demographic profile of patients with chest pain is summarized in Table 1.

**Table 1:** Demographic data of patients with chest pain.

Demographic characteristics		Number	Percentage (%)
Age in years (mean ± SD)		10.75 ± 2.47	
Gender	Male	26	47.3
	Female	29	52.7
Village/Town/City	Village	16	29.1
	Town	37	67.3
	City	2	3.6
Education of both parents	Graduation or above	36	65.5
	Others	19	34.5
Socioeconomic status	Upper and middle	45	81.8
	Lower	10	18.2

Chest pain of fewer than 48 hours, 48 hours to two weeks, and more than two weeks at presentation were found in six (10.9%), 11 (20%), and 38 (60.9%) children, respectively. It was found that 43 (78.2%) patients had screen time of more than two hours. Fourteen of the 43 children were used to screen viewing for over 10 hours. It was observed that 31 (56.4%) children had sedentary habits without outdoor play or daily exercise. Associated findings noted with chest pain were palpitation and breathing difficulty. Palpitation was recorded in 11 (20.4%) patients, whereas only four (7.3%) children had breathing issues. Characteristics of chest pain and associated findings are presented in Table 2.

**Table 2:** Chest pain characteristics and associated findings in children.

Characteristics		Number	Percentage (%)
Chest pain	<48 hours	6	10.9
	3–14 days	11	20.0
	>14 days	38	69.1
Screen time	≤2 hours/day	12	21.8
	>2 hours/day	43	78.2
Outdoor activity/Play	Absent	31	56.4
	Present	23	43.6
Palpitation	Absent	44	79.6
	Present	11	20.4
Respiratory distress	Absent	51	92.7
	Present	4	7.3

The etiological diagnosis for chest pain was made after a thorough evaluation and was broadly classified into organic, psychogenic, and idiopathic. To our surprise, chest pain was psychogenic in 83.6% of cases. Of 46 patients with non-organic/psychogenic chest pain, anxiety disorder (40%) and depression (21.8%) were the leading causes. Acute stress disorder (9.1%), conduct disorder (5.5%), dissociative disorder (3.6%), and panic disorder (3.6%) were other psychogenic causes. It is important to note that 13 (23.6%) children had associated smartphone and internet addiction. On comparing psychogenic causes of chest pain between males and females, we found no statistically significant difference (p = 0.72). Organic causes of chest pain were musculoskeletal, respiratory, cardiac, and gastrointestinal noted in six cases. One case of asthma and acute gastritis with chest pain was found. Cardiac abnormality in the form of anomalous origin of the right coronary artery was the sole cause of chest pain in our study. No definite etiology could be established in three cases with chest pain. A summary of the causes of chest pain is described in Table 3. Patient follow-up records, either physically or telephonically, were reviewed for at least six months. Of 55 children, 36 (63.6%) improved completely following appropriate therapy. Five children had some or no improvement in chest symptoms. No feedback or treatment outcome could be obtained from 15 (27.3%) children as they were lost to follow-up.

**Table 3:** Causes of chest pain in children (n = 55).

Etiology		Number (%)	Number (%)
Psychogenic	Anxiety disorder	22 (40)	46 (83.6)
	Depression	12 (21.8)	
	Acute stress disorder	5 (9.1)	
	Conduct disorder	3 (5.5)	
	Dissociative disorder	2 (3.6)	
	Panic disorder	2 (3.6)	
Organic	Musculoskeletal	3 (5.5)	6 (10.9)
	Respiratory	1 (1.8)	
	Cardiac	1 (1.8)	
	Gastrointestinal	1 (1.8)	
Idiopathic		3	3 (5.5)

## Discussion

Chest pain has always been considered a life-threatening symptom requiring urgent attention and referral to a pediatric cardiologist. Similarly, most patients included in our study were referred by a general practitioner or pediatrician for urgent attention. Because all patients in our study were evaluated with thorough history taking, clinical examination, and ECG by a pediatric cardiologist, there is only a small chance of any cardiac disorder being missed or misdiagnosed. Per our primary objective, we searched the etiology of these patients with chest pain. We found the cardiac cause in only one out of 55 patients. Anomalous origin of the right coronary artery was found by CT angiography of the coronary vessels in a 12-year-old boy referred to a higher center for surgical intervention. In our study, the organic cause was found in six (10.9%) patients. Among these causes, the musculoskeletal problem was found in three cases, one of asthma, and one of acute gastritis. Although the incidence of cardiac causes of chest pain is variable, it was found in 0-5% of children in previous studies [9]. Hanson et al. reported that 0.7% of children had cardiac causes of chest pain in their study [5]. Another study also reported cardiac causes of chest pain in 1% of children [10]. Musculoskeletal causes of chest pain include strain, trauma, costochondritis, and breast conditions. According to a few studies, musculoskeletal causes are the leading cause of chest pain, accounting for more than 50% of cases [11]. Respiratory causes of chest pain are usually easy to diagnose, including asthma, pneumonia, and others. Gastroesophageal reflux and acid peptic disorders are common gastrointestinal causes of chest pain [5].

Although the rare association of a cardiac disorder with chest pain is reassuring, psychogenic causes are a matter of concern. Psychogenic causes account for 10-30% of chest pain in children [11]. Based on previous studies, the frequency of idiopathic chest pain ranges from 20% to 61% [12-15]. Ayugun et al. found that 24.8% of patients with a psychogenic disorder with anxiety and depression are the most common comorbidities [16]. Various studies have consistently found anxiety as the leading psychiatric diagnosis in 50-85% of patients with non-cardiac chest pain [16,17]. In our study, 83.5% had psychogenic causes of chest pain. Only 5.5% of patients had no identifiable cause. This contrasts with previous studies where the idiopathic variety was much higher or similar to psychogenic causes. The higher frequency of psychogenic causes of chest pain in our study is possibly due to identifying possible stressors in all patients who would have ordinarily been categorized as idiopathic. In our study, anxiety disorder, smartphone and internet gaming addiction, and depression were three common psychiatric diagnoses, followed by acute stress disorder, conduct disorder, dissociative disorder, and panic disorder. Smartphone and internet addiction were significant risk factors in patients with chest pain. Notably, 78.2% of patients with chest pain had an average screen time of more than two hours daily. Associated smartphone and gaming addiction are directly or indirectly linked to sleep deprivation, stress, depression, anxiety, and multiple comorbidities affecting education and quality of life [18]. When examining the core issue for the above disorder, important factors such as failed relationships, family issues pertaining specifically to father and mother, poor interest in education, bad sleep habits, sedentary lifestyle, and screen dependence were likely triggering events in these children.

Chest pain as a presenting symptom is common in adolescents [9,19,20]. The mean age of presentation in our study was 10.75 ± 2.47 years. Males and females are almost equally involved, and there was no significant difference in the non-organic cause in both groups. Most studies have reported similar frequency and etiology of chest pain in both sexes [21-23]. We found a maximum number of patients from towns and villages rather than cities. In our study, the parents of these children were well-educated and belonged to middle and upper socioeconomic status. The probability of these children gaining access to more electronic gadgets and parental health-seeking might explain the higher frequency of chest pain in this group. Eighty percent of children in our study presented with chronic chest pain and continued chest pain for months has also been observed in previous studies [23,24]. Palpitation and respiratory distress were the only associated symptom in our patients observed in 20.4% and 7.3% of patients, respectively. In our study, 63.6% of patients had a resolution of pain after appropriate interventions. Unfortunately, 27.3% of our patients were lost to follow-up, and 9.1% of children had partial or no pain control. It signifies that addressing the non-organic cause in most patients will resolve their symptoms.

The limitation of this study is that it was a retrospective analysis of patients from records and involved a small number of patients. Our study had no comparison group, and many patients were lost to follow-up.

## Conclusions

Chest pain is one of the common complaints in the pediatric age group needing referral to a pediatric cardiologist. The most common etiology associated with chest pain is often non-cardiac and psychogenic. Good history taking and clinical examination with basic investigations, if required, are often sufficient to unravel the diagnosis in primary care settings. Efforts to exclude cardiac and other organic causes must be made in all children with chest pain before labeling them psychogenic. A large prospective controlled research is needed to establish the etiology and framework for diagnosing and managing chest pain in children.
